# Characterization of Ebola Virus Disease (EVD) in Rhesus Monkeys for Development of EVD Therapeutics

**DOI:** 10.3390/v12010092

**Published:** 2020-01-13

**Authors:** Travis Warren, Elizabeth Zumbrun, Jessica M. Weidner, Laura Gomba, Franco Rossi, Roy Bannister, Jacqueline Tarrant, Matthew Reed, Eric Lee, Jo Lynne Raymond, Jay Wells, Joshua Shamblin, Kelly Wetzel, Ginger Donnelly, Sean Van Tongeren, Nicole Lackemeyer, Jesse Steffens, Adrienne Kimmel, Carly Garvey, Holly Bloomfield, Christiana Blair, Bali Singh, Sina Bavari, Tomas Cihlar, Danielle Porter

**Affiliations:** 1 United States Army Medical Research Institute of Infectious Diseases, Frederick, MD 21702, USA; elizabeth.e.zumbrun.civ@mail.mil (E.Z.); jessica.m.weidner.ctr@mail.mil (J.M.W.); laura.m.gomba.ctr@mail.mil (L.G.); franco.d.rossi.ctr@mail.mil (F.R.); matthew.c.reed1.mil@mail.mil (M.R.); eric.d.lee2.mil@mail.mil (E.L.); jolynne.w.raymond.civ@mail.mil (J.L.R.); jay.b.wells.ctr@mail.mil (J.W.); joshua.d.shamblin1.civ@mail.mil (J.S.); kelly.s.wetzel2.ctr@mail.mil (K.W.); ginger.c.lynn.ctr@mail.mil (G.D.); sean.a.vantongeren.ctr@mail.mil (S.V.T.); nicole.l.lackemeyer.ctr@mail.mil (N.L.); jesse.t.steffens.ctr@mail.mil (J.S.); adrienne.l.kimmel.ctr@mail.mil (A.K.); carly.b.garvey.ctr@mail.mil (C.G.); holly.a.bloomfield.civ@mail.mil (H.B.); sina.bavari@icloud.com (S.B.); 2 Geneva Foundation, City, Tacoma, WA 98402, USA; 3 Laulima Government Solutions, Orlando, FL 32826, USA; 4 Gilead Sciences, Foster City, CA 94404, USA; roy.bannister@gilead.com (R.B.); Jacqueline.tarrant@gilead.com (J.T.); Chris.Blair@gilead.com (C.B.); bali.singh@gilead.com (B.S.); tomas.cihlar@gilead.com (T.C.); danielle.porter@gilead.com (D.P.)

**Keywords:** Ebola virus, animal model, EVD, disease, rhesus, macaque, monkey

## Abstract

Recent Ebola virus (EBOV) outbreaks in West Africa and the Democratic Republic of the Congo have highlighted the urgent need for approval of medical countermeasures for treatment and prevention of EBOV disease (EVD). Until recently, when successes were achieved in characterizing the efficacy of multiple experimental EVD therapeutics in humans, the only feasible way to obtain data regarding potential clinical benefits of candidate therapeutics was by conducting well-controlled animal studies. Nonclinical studies are likely to continue to be important tools for screening and development of new candidates with improved pharmacological properties. Here, we describe a natural history study to characterize the time course and order of progression of the disease manifestations of EVD in rhesus monkeys. In 12 rhesus monkeys exposed by the intramuscular route to 1000 plaque-forming units of EBOV, multiple endpoints were monitored for 28 days following exposure. The disease progressed rapidly with mortality events occurring 7–10 days after exposure. Key disease manifestations observed consistently across the infected animals included, but were not limited to, viremia, fever, systemic inflammation, coagulopathy, lymphocytolysis, renal tubular necrosis with mineralization, and hepatocellular degeneration and necrosis.

## 1. Introduction

Ebola virus (EBOV) is a human pathogenic virus and, along with Marburg and Sudan viruses, is a member of the Filoviridae family. First identified in 1976, outbreaks of EBOV have occurred sporadically, over a wide geographic area, in sub-Saharan Africa. The 2013–2016 outbreak in West Africa was the largest and most complex outbreak since EBOV was identified as the etiological agent of EBOV disease (EVD). More cases and deaths occurred in that outbreak than in all others combined. The World Health Organization (WHO) reported a total of 28,646 cases of EVD and 11,323 EVD-related deaths worldwide by the end of the outbreak [1]. In 2018, two independent outbreaks of EBOV were reported in the Democratic Republic of the Congo (DRC [formerly Zaire]); the larger of these two outbreaks, which is ongoing at the time of writing, has produced a total of 3,318 EVD cases, including 2,204 deaths [2].

The National Institute of Allergy and Infectious Diseases has sponsored the Pamoja Tulinde Maisha (PALM) randomized controlled trial (RCT) comparing ZMapp, remdesivir, mAb114, and REGN-EB3 (randomization = 1:1:1:1, N = 673) in patients with acute EVD. On 12 August 2019, the trial’s independent Data and Safety Monitoring Board recommended that the study design be altered due to promising results with two of the investigational agents [3]. The success of this study, and the demonstrated feasibility of obtaining robust clinical data, represents a paradigm shift in the regulatory processes by which the efficacy of current and likely future investigational therapeutics is to be characterized. Prior to this success, when the feasibility of obtaining efficacy data from a sufficient number of humans was in question, the Food and Drug Administration (FDA) Animal Rule (AR) provided a potential regulatory pathway to drug approval for EBOV therapeutics. However, no regulatory consensus or concurrence had been published regarding which, if any, animal model was adequate and well characterized, leaving investigators with many uncertainties regarding the experimental design, endpoints, and quality system required to meet these and other regulatory criteria. Nevertheless, given the technical complexities and uncertain timing of EBOV outbreaks, it can be expected that a nonclinical model will continue to serve as a useful tool in developing and/or refining new and existing antiviral candidates.

Several nonhuman primate (NHP) species—including African green monkeys, cynomolgus macaques, rhesus macaques, marmosets, and baboons—have been used as test systems in attempts to model human EBOV infection. Maculopapular rash, a feature of disease in humans, is a typical manifestation in macaques and baboons but not African green monkeys or marmosets [4,5]. Baboons present challenges in biocontainment experimental settings due to their larger size (requiring larger cage sizes) and shortage of available animals [6]. Cynomolgus and rhesus macaques have been the most widely used NHP species for characterizing disease manifestations following experimental filovirus inoculations and for nonclinical assessments of efficacy involving candidate vaccines or treatments. Historical conventions have prompted the use of cynomolgus macaques mostly for vaccine studies and rhesus macaques for therapeutic evaluations [7], although compelling scientific rationale supporting this historical bias is lacking.

Rhesus macaques have several advantages as a test species: (a) they share a close phylogenetic relationship with humans; (b) they can be infected by the same EBOV isolates that cause disease in humans; (c) key human EVD manifestations are recapitulated; and (d) they are sufficiently large to allow for repeated blood sampling to monitor multiple endpoints.

We conducted a controlled study, the overall goal of which was to evaluate the natural history of an EBOV infection in healthy, research-naïve rhesus macaques. The specific objectives of this study were to establish the following characteristics of disease in rhesus macaques exposed via intramuscular (IM) injection to EBOV relative to mock-infected control animals:

- Time from EBOV exposure to the onset of the manifestations of disease (e.g., signs, clinical and pathological features, laboratory parameters, extent of organ involvement, morbidity, and outcome)
- Time course and order of progression of the manifestations
- Frequency and severity of manifestations
- Individual or combined manifestations of EVD that would be of potential use as triggers for initiation of treatment in subsequent evaluations of investigational therapeutics using this disease model

The study contained two experimental arms, with *n* = 12 animals exposed to EBOV and *n* = 6 mock-exposed animals serving as controls. All 12 EBOV-exposed animals became infected and developed illness, and 11 of 12 EBOV-exposed animals (91.7%) succumbed within 7–10 days post inoculation (PI). We describe here manifestations of EVD in rhesus that could be used as triggers for initiation of treatment in future studies testing the safety and efficacy of investigational therapeutic agents in this model. Additionally, we provide a comparative analysis of human vs. NHP disease course and manifestations.

## 2. Materials and Methods

### 2.1. Quality System

The study was conducted in compliance with Good Laboratory Practices (GLPs) to enhance the likelihood that the data quality and integrity would comply with regulatory expectations. The FDA provided comments on the study design, and the authors incorporated the agency’s feedback into the final protocol.

### 2.2. Subjects and Experimental Design

Eighteen rhesus macaques (8 males and 10 females) were randomly assigned to either a mock-exposed group (Group 1, *n* = 6 [2 males, 4 females]) or an EBOV-exposed group (Group 2, *n* = 12 [6 males, 6 females]), stratified by sex and balanced by body weight. Animals in Group 2 were exposed to a target dose of 1000 plaque-forming units (pfu) EBOV in a 0.5-mL IM injection (431 pfu, calculated dose based on neutral red plaque assay of virus challenge stock material). Animals in Group 1 were administered 0.5 mL diluent (minimum essential medium with 2% [*v/v*] heat-inactivated fetal bovine serum) by the IM route and served as the mock exposure group. For this manuscript, the day of exposure for both groups is designated Day 0.

On Day 0, animals assigned to the mock-exposed group were inoculated first, to minimize the risk of potential virus exposure; animals in the EBOV-exposed group were inoculated immediately afterward. Right and left quadriceps were shaved, and the inoculation site was identified using a permanent marker to facilitate identification during necropsy. The inoculation site was in the same location for all animals (right quadriceps).

The schedule of study events is provided in Table A1.

### 2.3. Virus History, Propagation, and Characterization

The virus used in this study was Ebola virus H. sapiens-tc/ZAI/1995/Kikwit (order Mononegavirales, family *Filoviridae*, species *Zaire ebolavirus*). EBOV was obtained from a 65-year-old female patient during an outbreak occurring in 1995 in DRC. The patient exhibited disease, was hospitalized, and died. The US Centers for Disease Control and Prevention conducted the first passage of the virus (designated 807223) using Vero E6 cells. The University of Texas Medical Branch conducted a second passage of virus, designated WRC000121. The US Army Medical Research Institute of Infectious Diseases (USAMRIID) produced the passage-3 USAMRIID master seed stock, designated R4415, from WRC000121 using BEI-Vero E6 cells. The identity of this stock has been confirmed by agent-specific reverse-transcription polymerase chain reaction (RT-PCR) assay(s) as well as by sequencing on the Illumina MiSeq (150 bp paired-end format). This stock was determined to be 92.80% 7U variant; a genomic characterization of this stock was described by Kugelman et al. [8]. All propagation and characterization activities of the challenge agent stock occurred outside of the scope of this study.

### 2.4. Husbandry

Animals were housed one per cage in stainless steel cages with squeeze capabilities for handling. Primary enclosures conform to guidelines specified in the US Department of Agriculture Animal Welfare Act (9 CFR, Parts 1, 2, and 3) and as described in the 2011 Guide for the Care and Use of Laboratory Animals [9]. Research was conducted under an IACUC approved protocol in compliance with the Animal Welfare Act, PHS Policy, and other Federal statutes and regulations relating to animals and experiments involving animals. IACUC approval for the study protocol was obtained on 19OCT2017, with protocol amendment approvals obtained 20NOV2017 and 09JAN2018.

Mock-exposed animals were housed in an animal BSL-4 (ABSL-4) room separate from that of EBOV-exposed animals, but both groups were housed in the same ABSL-4 suite.

Animals were monitored by husbandry staff at least twice daily.

Animals were maintained on a 12-h light/12-h dark photoperiod, except when room lights were turned on for study-related procedures (e.g., animal observations). Temperatures were targeted between 18 °C and 29 °C with a relative humidity of 30–70%. Animals were provided ad libitum water and food (2050C Certified Monkey Diet [Envigo Teklad]); fruits and vegetables were also provided. Each NHP cage included an elevated perch or platform, and each NHP was provided with hard/soft toys for physical enrichment. For the single EBOV-exposed animal that survived the challenge, USAMRIID veterinarians developed an enhanced enrichment plan to reduce isolation stress. This animal received additional food enrichments at observation and routine husbandry events, was provided with audio stimulation by radio, and was given additional supplementary treat-filled toys to promote foraging behaviors. Staff were notified by a veterinarian to monitor this animal carefully for signs of stress behaviors and were encouraged to report findings consistent with stress behaviors.

Oral rehydration solution (Pedialyte^®^ mixed with water) was provided to all animals from the day of exposure onward. The volume of Pedialyte provided to each animal was evaluated once daily, and the volume remaining was documented in study records. Oral rehydration solution was included in the experimental design at the recommendation of the FDA during protocol review, to encourage voluntary oral fluid intake and reduce risk of dehydration.

### 2.5. Surgical Procedures

Prior to each surgical procedure, a veterinarian confirmed the health status of the animal. On the day of surgery, the animal was anesthetized and transported to the surgery location, and the surgical sites were prepared. Anesthesia was maintained during surgery.

To acquire body temperature and activity data, telemetry DSI M00 (Data Sciences International) was surgically implanted into each animal 36 days prior to exposure. The transmitter was implanted in a pocket created underneath the external abdominal oblique muscle.

To allow for venous access without use of anesthesia, a jugular central venous catheter (CVC) was surgically implanted into each animal 16–19 days prior to exposure and after 3 days of acclimation to Lomir jacket and tethering. During this procedure, a single-lumen broviac CVC (6.6 Fr) was inserted into a subcutaneous tunnel created underneath the neck muscles, over one shoulder, and to the exit location in the center of the back. After closure of the last skin incision, the NHP was transported to radiology to confirm correct placement of the catheter.

### 2.6. Inclusion/Exclusion Criteria

Animal health evaluations prior to study start included a physical exam, behavioral assessment, and evaluation of clinical pathology parameters. In addition, animals were subjected to health screens within 6 months prior to the initiation of jacket acclimation to ensure that they met the following inclusion criteria:

- Negative retroviral screening tests (STLV-1; SIV; and SRV-1, -2, and -3)
- Negative PCR and immunoglobulin G (IgG) screening tests for *Trypanosoma cruzi*
- Negative herpes B test
- The final of three consecutive negative tuberculosis tests
- Stool cultures negative for *Salmonella*, *Shigella*, and *Campylobacter*

Exclusion criteria were as follows:

- Under treatment for an existing disease condition or injury within 30 days prior to study initiation
- History of gastrointestinal disorders requiring treatment within 30 days prior to study initiation
- Preexisting antibodies (IgG) to EBOV glycoprotein (GP)

### 2.7. Experimental Blinding

Because Group 1 and Group 2 animals were housed in separate rooms, it was not possible to experimentally blind study staff to the group assignment of animals.

### 2.8. Clinical Observations

Prior to challenge, baseline observations were performed by trained personnel to document individual animal behaviors and appearance. (These observations were in addition to the routine observations conducted by husbandry staff.) Animals were observed once daily from Day −4 (4 days prior to inoculation) to Day 4 PI. At each observation, animals were assigned a responsiveness score using a five-point scale, as follows: 0 (alert, responsive, normal species-specific behavior), 1 (slightly diminished general activity, subdued, but responds normally to external stimuli), 2 (withdrawn, may have head down, upright fetal posture, hunched, reduced response to external stimuli), 3 (prostrate but able to rise if stimulated, or dramatically reduced response to external stimuli), and 4 (persistently prostrate, severely or completely unresponsive).

At the first observation on Day 5 PI, one animal in the EBOV-exposed group was assigned a responsiveness score = 1, prompting an increase in the frequency of observations to five per day for all animals, per protocol. On Day 11 PI, when the responsiveness score for all remaining animals (including the single EBOV-exposed survivor) had returned to 0, once-daily observations resumed; once-daily observations were continued through the end of the in-life phase.

Physical examination required animals to be anesthetized. Physical examination of animals under anesthesia occurred after unanesthetized observations on Day −4; Day 0 (prior to exposure); on Days 14, 21, and 28 PI; and when an animal succumbed or was euthanized or if catheter troubleshooting procedures required anesthesia of an animal. Other than scheduled anesthetized physical exams, all attempts were made to minimize instances in which animals were anesthetized.

Unanesthetized animals were evaluated cage-side for signs of illness, including but not limited to: cough, swelling, rash, bleeding, and impaired motor function. Other observations, such as biscuit/fruit consumption, condition of stool, and urine output, were also documented when possible.

### 2.9. Euthanasia

To be declared moribund, an animal had to be assigned a responsiveness score of 4 (persistently prostrate, severely or completely unresponsive). Euthanasia was conducted by intracardiac or CVC administration of a pentobarbital-based euthanasia solution (0.3–0.4 mL/kg) under deep anesthesia.

### 2.10. Necropsy and Post-Mortem Analyses

Unscheduled necropsies (on animals that were moribund or found deceased) were performed as soon as feasible following euthanasia or after they were found deceased, generally within 12 h of death. Necropsies on all animals were conducted by a board-certified veterinary pathologist in the BSL-4 suite. The necropsy included examination of the carcass and musculoskeletal system; all external surfaces and orifices; the neck with associated organs and tissues; and cranial, thoracic, abdominal, and pelvic cavities with their associated organs and tissues.

Tissues and organs were first examined in situ, and then were dissected from the carcass. Specimens were collected and fixed by immersion in labeled containers of 10% neutral buffered formalin. All collected tissues were documented, including additional tissues collected at the discretion of the pathologist and/or study director. All gross findings were recorded per individual animal in descriptive terms, including location(s), size, shape, color, consistency, and number as appropriate. All tissues with significant gross lesions were also collected and recorded. All necropsy and tissue collection data were maintained in Pristima (version 7.3) at USAMRIID.

The tissue samples were trimmed, routinely processed, and embedded in paraffin. After the paraffin-embedded tissues were cut and placed on glass slides, they were deparaffinized, stained with hematoxylin and eosin, and coverslipped. Replicate tissue sections were placed on positively charged slides and stained for immunohistochemistry (IHC) using a cocktail of mouse monoclonal anti-EBOV antibodies (USAMRIID #702/703) for detection of viral products VP40 and GP. To detect EBOV, genomic RNA was detected using in situ hybridization (ISH) in formalin-fixed, paraffin-embedded tissues, using the RNAscope 2.5 HD RED kit according to the manufacturer’s instructions.

### 2.11. Telemetry

Body temperature and activity were monitored by telemetry. Digital data were captured, reduced, and stored in telemetry data files (i.e., NSS files) using the Notocord-hem Evolution software platform (Version 4.3.0.75, Notocord Inc., Newark, NJ). Data in the NSS files were extracted and further reduced into a validated Microsoft Excel workbook (USAMRIID Excel Template V1.0) for each macaque using Notocord-derived formula add-ins. Data reduction was done in 30-min intervals for temperature and 6- and 12-h intervals for animal movement/activity. NSS data files were generated during data acquisition to record at least 5 days of baseline prior to virus or mock exposure. Fever was defined as body temperature > 1.5 °C above time-matched baseline for longer than 2 h. Hyperpyrexia was defined as body temperature > 3.0 °C above time-matched baseline for longer than 2 h. Severe hypothermia was defined as body temperature > 2.0 °C below time-matched baseline for 30 min.

### 2.12. Clinical Pathology

Clinical pathology assessments were conducted on all animals to monitor progression of pathophysiological endpoints at multiple time points and at the time of euthanasia. Blood was collected from nonfasted animals via CVC (or venipuncture for catheters lacking patency) for hematology, coagulation, and clinical chemistry analyses in accordance with the study schedule (Appendix A). On scheduled blood collection days, blood collection occurred following the first morning observation. For terminal blood collections, blood was collected by cardiac puncture or CVC under deep anesthesia immediately prior to euthanasia.

#### 2.12.1. Hematology

Blood samples were transferred to EDTA tubes at the time of collection. Hematology analysis was conducted using an Advia 120 Hematology Analyzer (Siemens) with multispecies software. The following hematologic parameters were analyzed: neutrophils, lymphocytes, monocytes, leukocytes, eosinophils, basophils, red blood cell count (RBC), hemoglobin (Hgb), hematocrit (Hct), mean corpuscular volume, mean corpuscular Hgb, mean corpuscular Hgb concentration, red cell distribution width, platelet count, mean platelet volume, and reticulocyte count.

#### 2.12.2. Coagulation Analysis

Blood samples were transferred to 3.2% sodium citrate tubes at the time of collection. Samples were processed to plasma and analyzed using a Sysmex CA-1500. The coagulation parameters analyzed included: prothrombin time (PT), activated partial thromboplastin time (APTT), fibrinogen, thrombin time, D-dimer, and antithrombin.

#### 2.12.3. Serum Chemistry

Blood samples were transferred to serum tubes at the time of collection. Blood was processed to serum and was analyzed using a VITROS 350 Chemistry System (Ortho Clinical Diagnostics). The following serum chemistry parameters were analyzed: sodium, potassium, chloride, alanine aminotransferase (ALT), aspartate aminotransferase (AST), alkaline phosphatase (ALP), gamma glutamyl transferase (GGT), lactate dehydrogenase, conjugated and unconjugated bilirubin, calcium, creatine kinase, blood urea nitrogen (BUN), creatinine, total protein, albumin, globulin, glucose, C-reactive protein (CRP), and carbon dioxide (CO_2_).

### 2.13. Viral Load Analyses

#### 2.13.1. Plasma Viral RNA

Validated RT-PCR methods were used for the quantification of the systemic (plasma) concentration of viral RNA in samples collected at the indicated times. Briefly, TRIzol LS-inactivated samples were extracted and eluted with AVE buffer using a QIAamp Viral RNA Mini Kit. The RT-PCR reaction used the SuperScript II One-Step RT-PCR System (Invitrogen) with additional MgSO_4_ added to a final concentration of 3.0 mM. Samples were run on an ABI 7500 Fast Dx, and data were acquired and analyzed using ABI 7500 FAST SDS v1.4. Samples for which ≥ 2 replicates were below the limit of detection (LOD, Ct = 38.07) of the assay were interpreted and reported as “<LOD” for EBOV RNA in plasma. Samples for which the analyzed value was above the LOD but below the lower limit of quantitation (LLOQ) were interpreted and reported qualitatively only (as “>LOD, <LLOQ”). Samples for which the reported value was above the LLOQ were reported quantitatively. The LOD for rhesus is 38.07 cycle threshold, and the LLOQ is 8 × 10^4^ ge/mL.

#### 2.13.2. Serum Infectious Virus

Plaque assay was used to assess the burden of infectious virus in serum collected at multiple time points. Briefly, frozen serum was thawed at ambient temperature and diluted using a 10-fold dilution series, starting with a 1:50 dilution, in Eagle’s essential minimal medium containing cell culture supplements. Vero cell monolayers in six-well plates were treated with 100 µL of diluted serum in duplicate for 1–2 h, after which cells were overlaid with EBME-containing agarose. Plates were incubated under standard cell culture conditions for 8 days to allow for plaque formation. Secondary agarose overlay containing neutral red was applied to each well for 23–24h to aide in plaque visualization and enumeration. Determination of pfu per volume of serum was conducted for each sample using the plaque number obtained from the least dilute serum sample for which plaques could be counted. Plaque counts < 10 and > 150 per well were considered unreliable and were excluded from analysis. The LOD for this plaque assay, starting with a 1:50 dilution of the serum and plating 100 µL per well in four wells (six-well dish format), is 2.10 log_10_ pfu/mL (125 pfu/mL), equivalent to 1 pfu detected in one of four wells. The LLOQ for the plaque assay is 3.10 log_10_ pfu/mL (1250 pfu/mL), equivalent to 10 pfu detected in one of four wells. In samples in which plaques were observed in one or more replicate wells, at counts < LLOQ, the calculated concentration of infectious virus was considered to be unreliable and was designated DNQ (detectable not quantitative).

### 2.14. Serum Soluble EBOV Glycoprotein Assessment

The concentration of EBOV soluble GP (sGP) was measured in serum samples via enzyme-linked immunosorbent assay (ELISA [Integrated BioTherapeutics, Bethesda, MD]). The assay was conducted according the manufacturer’s directions. Plates were read by determining the optical density of each well at a wavelength of 650 nm in a microtiter plate reader (BioTek ELX808 with Gen 5 2.07 software). A standard curve was calculated and the EBOV sGP concentration of each test sample was determined. The LLOQ for the sGP assay was defined as the least concentrated nonzero standard on the standard curve across all plates used for this assay. Based on this criterion, the matrix LLOQ was designated 32.80 ng/mL. An LOD has not been established for this assay.

### 2.15. Statistical Analysis

Survival was analyzed using Fisher’s exact test (survival at Day 28 PI) and using the Kaplan–Meier method and log rank test (survival over time). Virological and disease sign endpoints were analyzed by Wilcoxon rank sum test or Fisher’s exact test for between-group comparisons and Wilcoxon signed rank test for within-group comparisons. No multiple comparison adjustments were applied.

Time-matched telemetry body temperatures and activity (animal movement within cage) at baseline, post-baseline, and change from baseline were summarized by day PI using descriptive statistics by exposure group. Comparisons of EBOV-exposed and mock-exposed animals were made using the Wilcoxon rank sum test for the time-matched value and time-matched change from baseline for each day PI. Comparisons of the time-matched changes from baseline within an exposure group were made using the Wilcoxon signed rank test for each day PI. In conducting analyses of body temperature and activity results from individual animals, determination of statistical significance was based on temperature values that were 3 standard deviations (SD) above or below their concomitant time-matched baseline value.

Differences between groups or from baseline were considered to be statistically significant at an α = 0.05.

## 3. Results

### 3.1. Survival

**Figure 1:**
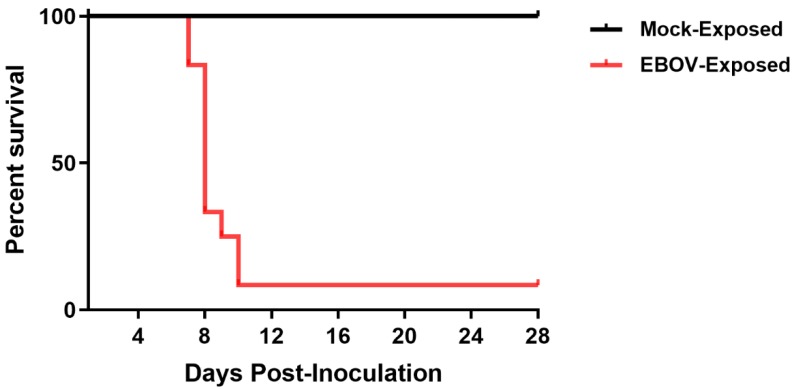
Kaplan–Meier plot of survival of EBOV-exposed and mock-exposed rhesus macaques.

All Group 1 (mock-exposed) animals survived to the end of the in-life phase of the study. Of the 12 Group 2 (EBOV-exposed) animals, 11 (91.7%) succumbed during the study, with mortality events occurring on Days 7–10 PI (Figure 1). Ten of these animals were euthanized after meeting protocol-specified euthanasia criteria (i.e., assignment of a responsiveness score = 4). Animals were euthanized on Days 7 (*n* = 2), 8 (*n* = 5), 9 (*n* = 1), and 10 PI (*n* = 2). One Group 2 (EBOV-exposed) animal was found deceased on Day 8 PI at 00:04, 6.35 h after the last documented unanesthetized observation. All mortalities are attributed to EBOV exposure. One Group 2 (EBOV-exposed) animal survived to the end of the in-life phase of the study.

In animals that succumbed, the average survival time (time from virus exposure to assignment of a responsiveness score = 4, or to the time found deceased) was 201.5 h (8.40 days PI) with a range of 179.9–242.5 h (7.50–10.10 days PI).

### 3.2. Clinical Observations

#### 3.2.1. Responsiveness Score

**Figure 2:**
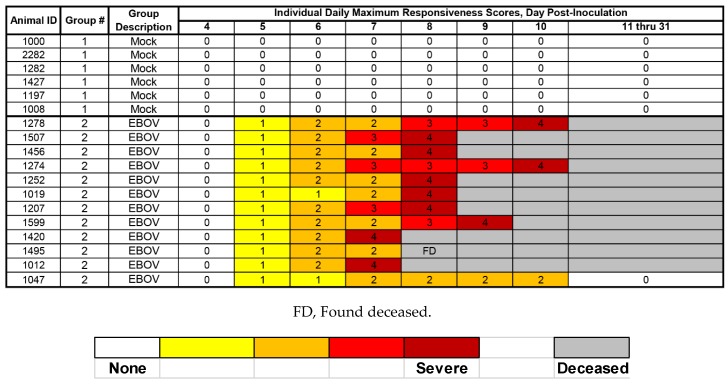
Daily maximum responsiveness scores.

Each NHP in Group 1 (mock exposure) was assigned a responsiveness score = 0 at all observations (Figure 2) and appeared healthy at all cage-side observations throughout the study. Onset of clinically observed reduced behavioral activity and change in posture (i.e., assignment of a responsiveness score ≥ 1) first occurred on Day 5 PI; on this day, all EBOV-exposed (Group 2) animals were assigned a responsiveness score = 1 during at least one observation event (Figure 2). The average time between exposure to virus in the EBOV-exposed group and assignment of a responsiveness score of 1 was 122.2 h (5.09 days PI), with a range of 116.1–126.9 h (4.83–5.29 days PI). In animals that succumbed, the average time between assignment of a responsiveness score = 1 and assignment of a responsiveness score = 4 or time found deceased, was 78.7 h (3.28 days PI), with a range of 55.3–119.1 h (2.30–4.96 days PI).

#### 3.2.2. Rash

Rash was not observed in any mock-exposed animal at any time point. In contrast, rash was observed in all EBOV-exposed animals with onset occurring on Day 5 PI in four animals, and on Day 6 PI in the remaining eight animals. The average time between EBOV exposure and first observation of rash in individual animals was 139.3 h (5.80 days), with a range of 131.9–147.0 h (5.50–6.13 days). In the Group 2 (EBOV-exposed) survivor, rash resolved by Day 11 PI. Importantly, the presence of the catheter jacket prevented staff from observing rash on the torso of animals during cage-side (unanesthetized) observations. All observations of rash are attributed to EBOV exposure.

#### 3.2.3. Food Intake and Body Weight

All EBOV-exposed animals exhibited reduced food intake with onset on Day 5 or 6 PI (with only two exceptions where reduced intake was observed once prior to challenge for one animal and once on Day 3 PI for one animal). Additionally, 11 of 12 EBOV-exposed animals had reduced fruit intake with onset between Days 6 and 8 PI. The EBOV-exposed survivor began eating normally by Day 11 PI.

At the terminal exam, neither mock-exposed nor EBOV-exposed animals as a group had statistically significant changes in weight compared to baseline.

#### 3.2.4. Urine and Stool

The presence of urine output was documented at the first observation of each day in all animals except for three instances. One EBOV-exposed animal had no urine output observed on Day 9 PI, the day before that animal was euthanized. Additionally, two EBOV-exposed animals had no urine output on either Day 8 or Day 10 PI, corresponding to the times at which each of these animals was assigned a responsiveness score of 4. Stool appeared normal in all mock-exposed animals throughout the study. All EBOV-exposed animals developed some abnormality to the stool (no stool or liquid stool). For 11 of 12 EBOV-exposed animals, “no stool” was noted at various times from Day 6 to Day 11 PI.

### 3.3. Viral Load

#### 3.3.1. Plasma Viral RNA

**Figure 3:**
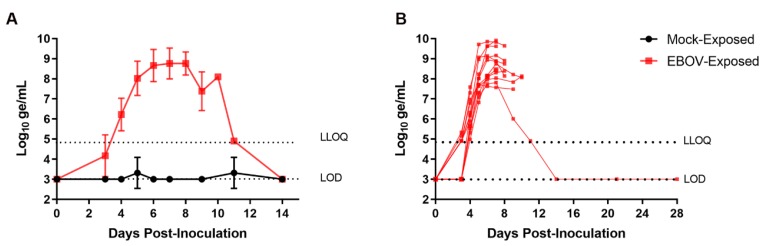
Group mean and individual animal plasma viral RNA: (**A**) group mean ± SD of plasma viral RNA versus time; and (**B**) plasma viral RNA concentration in individual EBOV-exposed animals versus time. LOD, limit of detection, Ct = 38.07; LLOQ, lower limit of quantitation = 4.903 log_10_ ge/mL. For display and analyses, EBOV RNA values below the LOD were imputed as 3.000 log_10_ ge/mL; values above the LOD but below the LLOQ were imputed as 4.903 log_10_ ge/mL. Statistical analyses were not performed.

RT-PCR for EBOV RNA was performed on plasma samples collected from each animal at intervals from Day −4 to Day 28 PI. Figure 3 shows group mean plasma viral RNA as well as plasma viral RNA for individual EBOV-exposed animals over the course of the study. Viral RNA was not detected at quantitative values in any sample from the mock-exposed group at any time during the study. Viral RNA was first detected in plasma on Day 3 PI in 7 of 12 EBOV-exposed animals (58%). At this time, four of these animals had viral RNA that was detectable but not quantitative, and three had quantitative results. On Day 4 PI, viral RNA was detected at quantitative concentrations in 12 of 12 EBOV-exposed animals (100%). Once detected, plasma viral RNA concentration generally increased rapidly in individual animals through Day 6 PI, with multiple instances of multi-log_10_ increases occurring between daily sampling events. Peak values in individual animals ranged from 7.71 to 9.91 log_10_ ge/mL and occurred on Days 5 (*n* = 1), 6 (*n* = 4), 7 (*n* = 5), 8 (*n* = 1), and 10 PI (*n* = 1). Values did not fall by more than 1 log_10_ ge/mL from the peak to the time when the nonsurviving animals succumbed.

For the surviving EBOV-exposed animal, systemic viral RNA was not detected until Day 4 PI, and this animal generally had lower overall viral burden than the other EBOV-exposed animals. On Days 4–7 PI, the surviving EBOV-exposed animal had viral RNA values among the lowest of EBOV-exposed animals and had the lowest viral RNA concentration of the four animals surviving to Day 9 PI. Maximum viral RNA concentration in this animal (7.81 log_10_ ge/mL) was obtained on Day 7 PI. Plasma viral RNA in this animal, while detectable on Day 11 PI, had resolved with no detectable virus in samples obtained on Day 14 PI and later.

#### 3.3.2. Serum Infectious Virus

Serum infectious virus was assessed by plaque assay on samples obtained on Day −4; Days 5, 7, 9, and 28 PI; and at the time of euthanasia. This sparse sampling strategy was necessitated based on limits to the volume of blood that could be collected and the desire to prioritize other endpoints. The presence of infectious virus was not observed for any serum samples collected from mock-exposed animals at any time point (Table 1). For serum samples collected from EBOV-exposed animals, infectious virus was detected on Day 5 PI in 10 of 12 animals. However, for six of these animals, the levels of virus, while detected, were below the LLOQ. The peak levels of virus in individual animals were observed on Days 7 (*n* = 6), 8 (*n* = 4), 9 (*n* = 1), and 10 PI (*n* = 1). The highest levels of viremia typically occurred at the final stage of disease for nonsurviving animals. For 8 of 11 nonsurviving animals, the peak value was the final value obtained, and, for the remaining three animals, subsequent data points did not decline by more than 0.2 log_10_ pfu/mL. The surviving EBOV-exposed animal had values in the lowest quartile on Day 7 PI and no infectious virus detected on Days 9 or 28 PI, aligning with recovery.

Importantly, results obtained from the assay used to quantitate infectious virus are expected to be affected by the presence of neutralizing antibodies that might be present in the samples. As a result, these data should be interpreted with caution.

### 3.4. Serum Soluble EBOV Glycoprotein

EBOV sGP was evaluated by ELISA on samples obtained on Day 0 (prior to mock and virus exposures); Days 4, 6, 11, 21, and 28 PI; and at the time of euthanasia (Table 2).

EBOV sGP concentrations did not achieve quantifiable levels in any sample obtained from mock-exposed animals at any time point. In EBOV-exposed animals, serum sGP concentrations ranged from 52.37 to 2703.20 ng/mL. Peak sGP values were the last values obtained for 10 of 11 nonsurviving animals. The EBOV-exposed survivor had the lowest level of sGP among the EBOV-exposed animals on Day 4 PI, and was in the lowest quartile on Day 6 PI. The peak sGP value for the surviving EBOV-exposed animal, occurring on Day 6 PI, exceeded the peak value of only one nonsurviving animal. Soluble GP was still present in the serum of the EBOV-exposed survivor on Day 11 PI, as the animal was recovering, but was <LLOQ on Days 21 and 28 PI.

### 3.5. Body Temperature Assessment by Telemetry

**Figure 4:**
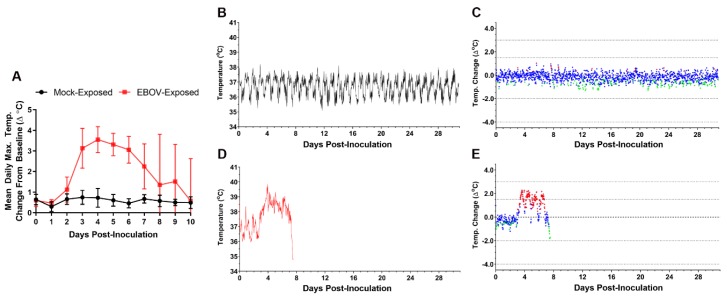
Telemetry-based body temperature changes in mock- vs. EBOV-exposed animals. (**A**) Group mean of daily maximum change from baseline body temperature. Error bars represent SD. X-axis has been truncated to highlight responses occurring from the time of mock or virus exposure events through the acute disease phase. No statistical analyses were performed. Representative mock-exposed animal absolute body temperature (**B**) and change from baseline (running 30-min average) (**C**). Representative EBOV-exposed animal absolute body temperature (**D**) and change from baseline (running 30-min average) (**E**). (**C**,**E**) Values −3 SD (♦) or +3 SD (♦) from baseline are statistically significant; values < 3 SD (♦) are not significant.

Body temperature was monitored in real time using biotelemetric monitoring. Figure 4 shows body temperature changes from baseline for a representative mock-exposed animal and an EBOV-exposed animal. The EBOV exposed animal was selected as representative because the time at which fever occurred in this animal was most closely matched to the mean for this group.

Five of six of the mock-exposed animals showed no sustained period of significant temperature increases during the study.

EBOV-exposed animals had sustained increases in body temperature with significant increases from baseline beginning an average of 3.22 days post exposure (range 2.56–4.10 days). All EBOV-exposed animals developed sustained fever on or before Day 4 PI (an average of 3.38 days [range, 2.63–4.31 days] after virus exposure), with 11 of 12 exhibiting fever on or before Day 3 PI. Statistically significant increases in body temperature of EBOV-exposed animals compared to baseline were observed in nearly all 30-min intervals on Day 3 PI, and there were multiple instances in which average change from baseline exceeded 2 °C. Fever coincided with an apparent alteration of diurnal rhythm for all EBOV-exposed animals (Figure 4D). Sustained hyperpyrexia (>3.0 °C above time-matched baseline for longer than 2 h) occurred in 9 of 12 animals starting at an average of 4.24 days (3.42–5.50 days) after exposure. Eight of 11 nonsurviving animals exhibited a significant decrease in body temperature prior to succumbing or euthanasia, starting an average of 8.33 days (range 7.17–10.06 days) after exposure. In the surviving EBOV-exposed animal, fever peaked at approximately Day 4 PI, followed by a gradual return to baseline temperature and normal diurnal rhythm by approximately Day 17 PI.

### 3.6. Activity Assessment Using Telemetry

Animal movement/activity was monitored in real time using telemetry and data that were summarized in 6- and 12-h intervals. The 12-h intervals were aligned with the 12-h light/dark cycle of the laboratory. Because animal activity was generally subdued during the dark cycle, discussion and analyses of activity results focus on the light period intervals.

**Figure 5:**
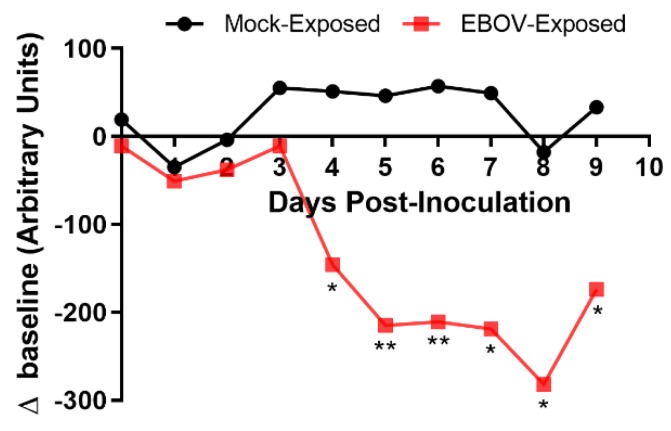
Group means of 12-h light period activity (movement in cage). Twelve-hour light period activity monitoring by telemetry occurred from 06:00 to 18:00. * *p* < 0.05; ** *p* < 0.001. *p*-values are indicated for comparison of change-from-baseline values in mock- vs. EBOV-exposed animals on the indicated study day.

In the mock-exposed animals, activity assessed using telemetry showed either slight decreases (<10% change from baseline) or increases from baseline during the light periods (06:00–18:00) as the study progressed (Figure 5).

Six-hour change-from-baseline activity values on Days 3 PI (12:00–18:00) and 4 PI (06:00–12:00) were significantly reduced in the EBOV-exposed group compared to the mock-exposed group. Uniform reductions in change-from-baseline activity values in all EBOV-exposed animals occurred beginning on Day 4 PI in the 12-h interval 06:00–18:00 and in the 6-h interval 12:00–18:00; these values were statistically significantly reduced compared to mock-exposed animals.

After the initial decline in light period activity of the EBOV-exposed survivor, activity of this animal remained depressed until approximately Day 18 PI, after which a gradual upward trend in light period activity, lasting until the end of the study, was recorded.

### 3.7. Clinical Pathology

The clinical pathology data for the EBOV-exposed group are diagnostic of severe systemic inflammatory response and disseminated intravascular coagulopathy (DIC) leading to damage and dysfunction of multiple tissues and organs, fluid loss that compounds organ dysfunction, and ultimately the terminal morbid state.

**Figure 6:**
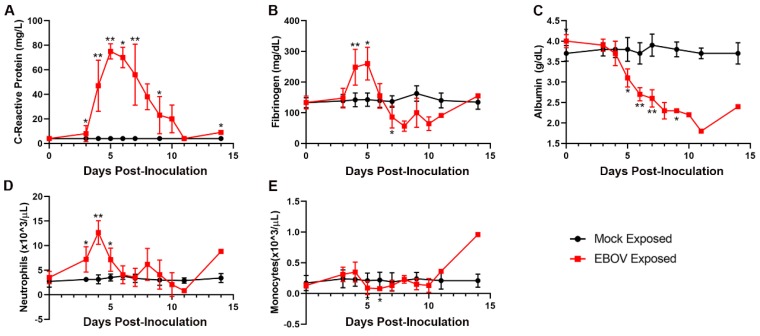
Exposure to IM EBOV in rhesus macaques produces clinical pathology alterations indicative of systemic inflammatory responses. Group mean ± SD of: C-reactive protein (**A**); fibrinogen (**B**); albumin (**C**); neutrophils (**D**); and monocytes (**E**). X-axes are truncated to highlight responses occurring from the first sampling point through the acute disease phase. * *p* < 0.05; ** *p* < 0.001. *p*-values are indicated for comparison of change-from-baseline values in mock- vs. EBOV-exposed animals on the indicated study day. C-reactive protein values < LLOQ (LLOQ = 5 mg/L) were assigned a value of 4 mg/L for display and analysis.

Changes in multiple study endpoints in the EBOV-exposed group—including, but not limited to, acute-phase proteins CRP, fibrinogen, and albumin as well as neutrophil and monocyte counts—are compatible with a severe systemic inflammatory response (Figure 6). CRP was markedly increased starting on Day 3 PI and peaked by Day 5 PI (14–24-fold baseline) and then declined, although CRP was still moderately to markedly elevated on the day of disposition. Albumin, a negative acute-phase protein, had a progressive, moderate to marked decrease over the course of the study, beginning from Day 4 PI. This decrease from baseline was statistically significantly different from the mock-exposed group on Days 4, 5, 6, 7, and 9 PI. Mean neutrophil counts were significantly increased versus baseline values on Days 3–5 PI. Notably, neutrophil counts decreased later in the course of the study in some animals, indicative of consumption of neutrophils that likely overwhelmed granulopoiesis by the bone marrow. Changes in neutrophil count correlated with the histologic findings of inflammation in the liver, inoculation site, and small intestine in several animals. Decreased lymphocyte counts occurring on Days 3–7 PI, had microscopic correlates of widespread lymphocytolysis and lymphoid depletion in the spleen, multiple lymph nodes, and the gut-associated lymphoid tissue of the intestinal tract. As lymphocytes are integral to regulating the adaptive immune response, this finding is compatible with a state of immunosuppression.

**Figure 7:**
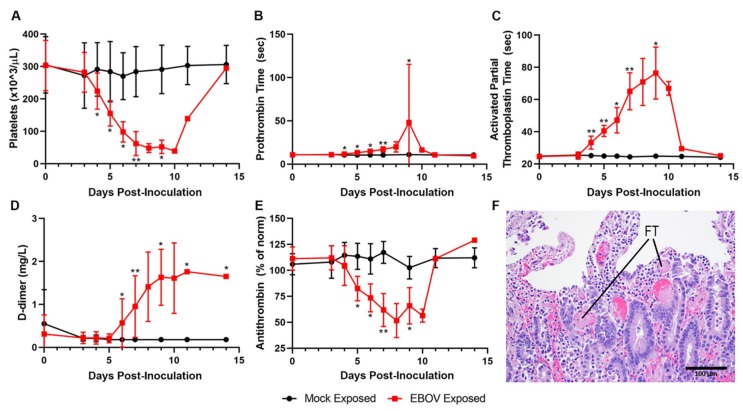
Exposure to IM EBOV in rhesus macaques produces clinical pathology and histological alterations indicative of coagulopathy, including disseminated intravascular coagulopathy. Group means ± SD of: platelets (**A**); prothrombin time (**B**); activated partial thromboplastin time (**C**); d-dimer (**D**); and antithrombin (**E**). X-axes are truncated to highlight responses occurring from the first sampling point through the acute disease phase. * *p* < 0.05; ** *p* < 0.001. *p*-values are indicated for comparison of change-from-baseline values in mock- vs. EBOV-exposed animals on the indicated study day. (**F)** Small intestine, duodenal mucosa showing numerous intravascular fibrin thrombi (FT, two representative examples noted).

Data from multiple coagulation endpoints—including decreased platelet counts, highly prolonged PT and APTT, increased D-dimers, and decreases in fibrinogen and antithrombin (Figure 7A–E)—are consistent with consumptive coagulopathy. Together with histopathologic evidence of intravascular fibrin thrombi in multiple tissues, these findings are diagnostic of DIC (Figure 7F). Statistically significant changes consistent with coagulopathy were noted beginning on Day 4 PI with changes usually progressing in severity over time.

**Figure 8:**
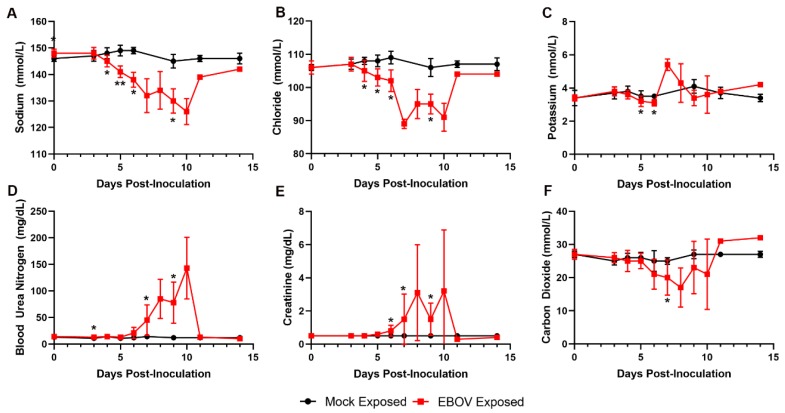
Exposure to IM EBOV in rhesus macaques produces clinical pathology alterations indicative of fluid loss. Group means ± SD of: sodium (**A**); chloride (**B**); potassium (**C**); blood urea nitrogen (**D**); creatinine (**E**); and carbon dioxide (**F**). X-axes are truncated to highlight responses occurring from the first sampling point through the acute disease phase. * *p* < 0.05; ** *p* < 0.001. *p*-values are indicated for comparison of change-from-baseline values in mock- vs. EBOV-exposed animals on the indicated study day.

Key serum chemistry changes reflect important fluid loss that led to dehydration and compromised organ perfusion and contributed to multiple organ injury and dysfunction (Figure 8). Marked decreases in serum sodium and chloride and mild decreases in potassium concentrations are consistent with fluid loss from the vascular compartment due to body cavity effusions and gastrointestinal tract loss (evident by observations of loose stool). Azotemia, characterized by moderate to marked elevations in BUN and creatinine, is compatible with dehydration and renal functional impairment with microscopic correlates of acute renal tubular degeneration and necrosis. Later in the course of the study, increased serum potassium and low serum CO_2_ indicated a presumptive metabolic acidosis related to renal impairment and lactic acidosis due to reduced tissue perfusion, which likely contributed to morbidity.

**Figure 9:**
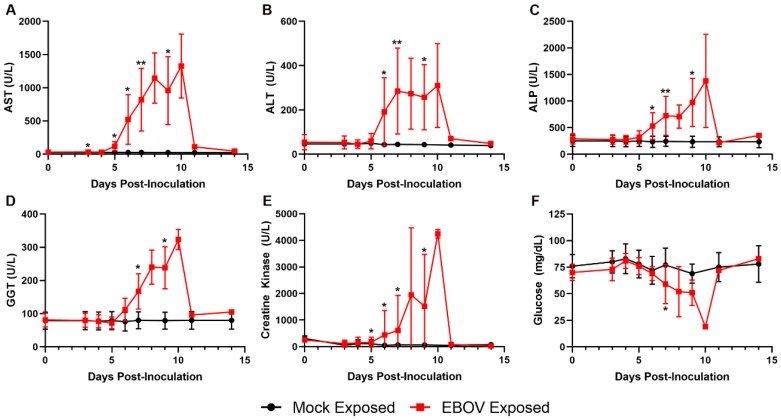
Exposure to IM EBOV in rhesus macaques produces clinical pathology alterations consistent with hepatocellular damage and necrosis and other disease conditions. Group means ± SD of: aspartate aminotransferase (AST) (**A**); alanine aminotransferase (ALT) (**B**); alkaline phosphatase (ALP) (**C**); gamma glutamyl transferase (GGT) (**D**); creatine kinase (**E**); and glucose (**F**). X-axes are truncated to highlight responses occurring from the first sampling point through the acute disease phase. * *p* < 0.05; ** *p* < 0.001. *p*-values are indicated for comparison of change-from-baseline values in mock- vs. EBOV-exposed animals on the indicated study day.

Increases in AST, ALT, ALP, and GGT in the EBOV-exposed group are attributed to hepatocellular damage and necrosis secondary to filoviral infection (Figure 9), as noted histologically in 11 of 12 animals. Increases in these liver enzymes were most prominent on Days 6 and 7 PI, with 5.3- and 38.4-fold mean increases over baseline for Day 7 PI ALT and AST, respectively. Increases in AST and ALT may have also resulted from muscle degeneration, which is further supported by observations of moderate to marked increases in creatine kinase at similar time points.

Glucose was decreased in 11 of 12 animals, often profoundly on the day of disposition (four animals had levels below the lower limit of the instrumentation). The decreased glucose was likely due to increased utilization as a result of severe systemic inflammation, hepatic impairment in some animals, and possibly terminal shock.

Although the EBOV-exposed survivor had a pattern of findings similar to those of nonsurvivors, this animal had notably less severe decreases in lymphocyte count, platelet count, antithrombin, and fibrinogen and less severe prolongation of PT, suggesting a lower grade of consumptive coagulopathy (data not shown). This animal also exhibited lesser changes in electrolytes, CO_2_, BUN, and creatinine, suggesting reduced fluid loss and metabolic and tissue dysfunction. The lower grade of these changes is compatible with a lower level of detectable virus and survival.

### 3.8. Anatomic Pathology

**Figure 10:**
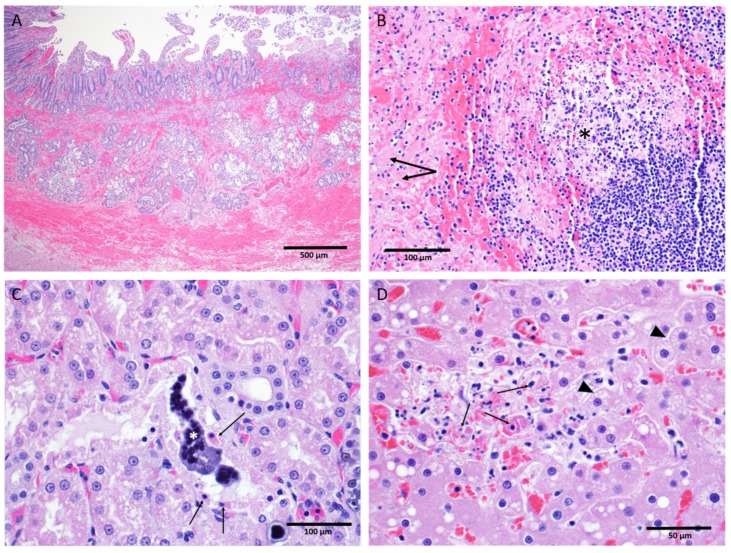
Salient microscopic findings in rhesus macaques exposed to IM EBOV. (**A**) Small intestine, duodenum: Diffuse hemorrhage expanding the mucosa and submucosa. (**B**) Spleen, white pulp: Lymphocyte depletion with lymphocytolysis (asterisk) and fibrin deposition (arrow) in the adjacent red pulp; (**C**) Kidney tubules: Necrosis of the tubular epithelium (arrows) with intratubular mineralization (asterisk). (**D**) Liver: An area containing hepatocellular degeneration (arrow heads) and necrosis (arrows).

Key gross and microscopic lesions included, but were not limited to, the following: macular skin rash, duodenal hemorrhage, lymphocytolysis, renal tubular necrosis with mineralization, splenic fibrin deposition, and hepatocellular degeneration and necrosis (Figure 10). All EBOV-exposed nonsurvivors had positive IHC and/or ISH results in liver, kidney, spleen, lung, heart, intestine, inoculation site, inguinal lymph node, male and female reproductive tissues, eyes, and other tissues.

#### 3.8.1. Gross Findings

The most consistent disease-related gross findings among the EBOV-exposed nonsurvivors were macular skin rash, duodenal mucosal hemorrhage (discoloration), cardiac petechial hemorrhages (discoloration), urinary bladder mucosal hemorrhage (discoloration), pale and/or friable liver (discoloration and abnormal consistency), large intestinal congestion (discoloration), splenic enlargement (increased size), dark red kidneys (discoloration), and dark red musculature of inoculation site (discoloration). The only gross observation recorded in the single EBOV-exposed survivor was lung discoloration that was also observed in mock-exposed animals and EBOV-exposed nonsurvivors. Lung discoloration was noted in 2 of 6 controls and 10 of 12 EBOV-exposed animals; this finding was most likely a combination of postmortem congestion and euthanasia artifact.

#### 3.8.2. Histological Findings

*EBOV antigen (IHC) and nucleic acid (ISH) observations.* All EBOV-exposed nonsurvivors had positive IHC and/or ISH results—indicating the presence of EBOV antigen or EBOV RNA, respectively—in liver, kidney, spleen, lung, heart, intestine, inoculation site, inguinal lymph node, male and female reproductive tissues, eyes, and other tissues. Mock-exposed animals were IHC and ISH negative across all tissues examined. The EBOV-exposed survivor had negative IHC and ISH staining across most tissues with the exception of the lung.

*Tissue-specific histological findings.* The skin and the inoculation site of the mock-exposed animals and the EBOV-exposed survivor appeared normal upon gross and microscopic examination. Macular skin rash was the most consistent gross finding among EBOV-exposed nonsurvivors, with 11 of 11 affected. Eight of 11 EBOV-exposed nonsurvivors had histopathology findings at the inoculation site.

No liver degeneration or necrosis was observed in the EBOV-exposed survivor. The liver of this animal was microscopically similar to those of the mock-exposed animals, with no inflammation or inclusions. In contrast, 5 of 11 EBOV-exposed nonsurvivors had pale and/or friable liver upon gross examination, 11 of 11 animals had mild to marked liver degeneration and necrosis, and 10 of 11 had notable inflammation (Figure 10D).

Overall, mock-exposed animals had normal spleen microscopically. However, the spleen of the EBOV-exposed survivor exhibited moderate lymphoid follicular hyperplasia. The spleen of all 11 EBOV-exposed nonsurvivors had numerous manifestations of disease, including lymphoid depletion, lymphocytolysis, marginal zone congestion, follicular hemorrhage within the white pulp, and fibrin deposition within the red pulp (Figure 10B).

The mock-exposed animals and the EBOV-exposed survivor had kidneys that generally appeared normal with the exception of mononuclear cell infiltrates in three mock-exposed animals. The kidneys of 11 of 11 EBOV-exposed nonsurvivors, however, displayed renal tubular degeneration, necrosis, mineralization, and fibrin thrombi (Figure 10C).

Hemorrhage of the duodenum and related gastrointestinal tract abnormalities were observed in 9 of 11 EBOV-exposed nonsurvivors (Figure 10A).

Nine of 11 EBOV-exposed nonsurvivors had lymphoid depletion in at least one lymph node; this was not observed in the mock-exposed animals or the EBOV-exposed survivor.

Three EBOV-exposed nonsurvivors had histologic lesions in the meninges, composed of meningeal congestion and/or diffuse mononuclear meningeal inflammation. The EBOV-exposed survivor had detectable inflammation in the brain itself, restricted to the perivascular space in the area of the thalamus.

No lesions were present in the ovaries. A single EBOV-exposed nonsurvivor had multifocal hemorrhage in the mucosa of the uterus. Testicular hemorrhage was present in all five male nonsurvivors. No other lesions were present in the testes.

### 3.9. Day-by-Day Summary of Disease Manifestations in the Rhesus IM/EBOV Disease Model

**Figure 11:**
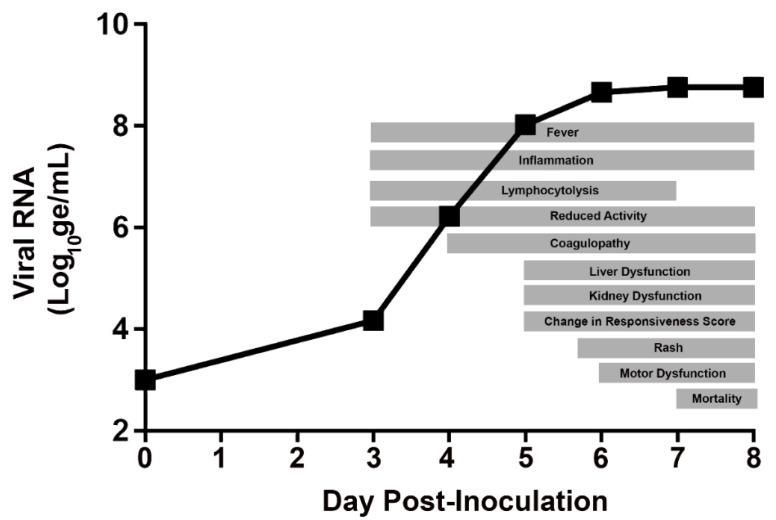
Schematic showing generalized progression of acute EVD following exposure to IM/EBOV in rhesus macaques. The day of EBOV exposure is designated Study Day 1.

This section presents a day-by-day summary of the observed kinetics of infection and of key disease manifestations in EBOV-exposed animals from the time of virus exposure to the terminal phase of disease. These changes are depicted in a schematic of generalized disease progression in Figure 11.

#### 3.9.1. Days 0–2 PI

No notable disease manifestations were observed in any study endpoint on Days 0–2 PI, except for a single instance of onset of fever (defined as body temperature > 1.5 °C above baseline for longer than 2 h) in one animal on Day 2 PI. No planned blood sampling events occurred on Days 1 or 2 PI, thus observations of disease signs were limited to telemetry-based endpoints (temperature and activity) and clinical observations.

#### 3.9.2. Day 3 PI

Multiple signs of EBOV disease first began on Day 3 PI. Although all animals appeared overtly healthy (i.e., were assigned a responsiveness score = 0) at all observation events, a statistically significant decrease in activity (as assessed by telemetry) occurred beginning on Day 3 PI. Onset of fever occurred in 11 of 12 animals on or before Day 3 PI, and onset of hyperpyrexia (defined as body temperature > 3.0 °C above baseline for longer than 2 h) occurred in 3 of 12 animals. Systemic viral RNA was first detected in 7 of 12 animals (this was the first day on which samples were collected for viral RNA analysis). Statistically significant changes from baseline were noted beginning in multiple clinical pathology parameters, supporting the onset of systemic inflammatory responses (elevated neutrophil and monocyte counts, and CRP) and decreased lymphocyte count.

#### 3.9.3. Day 4 PI

Although all animals were assigned a responsiveness score of 0 at all observation events, all animals showed a progressive reduction (compared to Day 3 PI) in telemetry-assessed activity (movement within cage). Onset of fever occurred in the final EBOV-exposed animal (the only animal of this group that had not previously developed fever), and hyperpyrexia was detected in 5 of 12 animals. Sustained temperature elevations occurred in all animals, with statistically significant elevations occurring in the majority—and, for some animals, all—of the daily 30-min temperature intervals for each animal. All animals also had detectable (and quantitative) systemic viremia with a mean viral RNA of 6.225 log_10_ ge/mL (range 4.980–7.590 log_10_ ge/mL), and all animals had detectable levels of sGP, ranging from 52.37 to 2703.20 ng/mL. Statistically significant changes in clinical pathology endpoints were indicative of greater severity of systemic inflammatory responses and lymphocytolysis. Statistically significant changes in coagulation parameters (e.g., prolonged PT and APTT and increased fibrinogen) were consistent with the beginning of coagulopathy.

#### 3.9.4. Day 5 PI

Onset of clinically observed reduced behavioral activity (i.e., assignment of a responsiveness score ≥ 1) first occurred on Day 5 PI, and all animals were assigned a responsiveness score = 1 during at least one observation event (Figure 2). Rash was first noted in four animals. Animals exhibited progressive decreases in activity levels compared to Day 4 PI (assessed by telemetry). Sustained temperature elevations continued in all EBOV-exposed animals. Logarithmic increases in mean viral RNA were observed from Day 4 to Day 5 PI in all EBOV-exposed animals with mean plasma viral RNA on Day 5 PI of 8.022 log_10_ ge/mL. Infectious virus was detected in serum in 10 of 12 animals. Changes in clinical pathology parameters were indicative of progressively more severe systemic inflammatory responses, lymphocytopenia, and coagulopathy (consumption of coagulation factors). Additional findings consistent with liver and kidney dysfunction were first noted on Day 5 PI.

#### 3.9.5. Day 6 PI

Clinically observed changes in behavioral activity and posture (i.e., assignment of a responsiveness score ≥ 1) worsened on Day 6 PI, with 10 of 12 animals assigned a responsiveness score of 2 on at least one observation event. Rash was observed in all animals. Animal activity levels (assessed using telemetry) were significantly decreased in all animals compared to baseline. Sustained temperature elevations continued in all EBOV-exposed animals, with statistically significant change-from-baseline elevations occurring in the majority of the daily 30-min temperature averages. Mean viral RNA began to plateau with a <1 log_10_-fold increase compared to Day 5 PI. Mean sGP levels (3148.24 ng/mL) increased relative to Day 4 PI. Inflammatory responses and lymphocytopenia were sustained without further worsening. Data from coagulation endpoints indicated progressively more severe coagulopathy. Indicators of red blood cell mass (Hgb, Hct, and RBC) were significantly reduced relative to Day 5 PI and remained low through the day of disposition. Rises in BUN and creatinine (azotemia) supported dehydration and possibly renal dysfunction, and increased liver enzymes (ALT, AST, ALP, and GGT) supported liver damage.

#### 3.9.6. Day 7 PI

Two animals succumbed on Day 7 PI, after having met euthanasia criteria (assignment of responsiveness score = 4). All remaining animals were assigned a responsiveness score ≥ 2 at all observation events, and 3 of 12 animals were assigned a responsiveness score of 3 on at least one observation event on this day. Decreases, compared to baseline, in telemetry-assessed animal activity levels remained statistically significant. Temperature elevations continued in the majority of animals, with statistically significant change-from-baseline elevations occurring in most time periods. In animals that succumbed, hypothermia preceded euthanasia. Mean viral RNA was 8.765 log_10_ ge/mL. Serum infectious virus was detected in all animals, with a mean of 4.80 log_10_ pfu/mL. Systemic inflammatory response was sustained. Lymphocyte counts began to recover. Reduction of fibrinogen and notably greater decreases in platelet count indicated consumption of key factors consistent with DIC. Azotemia and liver enzyme levels peaked on Day 7 PI. Additionally, disturbances in serum electrolytes and CO_2_ were consistent with severe dehydration from fluid loss and subsequent presumptive metabolic acidosis.

#### 3.9.7. Day 8 PI

Six animals succumbed on Day 8 PI, either having met euthanasia criteria (*n* = 5) or having been found deceased (*n* = 1). Of the remaining 4 animals, 3 animals were assigned a responsiveness score of 3 on at least one observation event, and the lone survivor in the group was assigned a score of 2. Animal activity levels (assessed using telemetry) remained significantly decreased compared to baseline. Temperature elevations generally continued in most animals. In animals that succumbed, occurrences of hypothermia preceded euthanasia. Lymphocyte counts continued to rebound, with increases relative to Day 7 PI observed for all animals. Clinical pathology endpoints were compatible with DIC, renal dysfunction, dehydration, and liver damage.

#### 3.9.8. Days 9 PI to End of In-Life

Three of the four remaining EBOV-exposed animals succumbed on Day 9 PI (*n* = 1) or Day 10 PI (*n* = 2), having met euthanasia criteria. In animals that succumbed, plasma viral RNA remained elevated with a mean at the time of euthanasia of 7.92 log_10_ ge/mL. In addition, in nonsurvivors, lymphocyte counts continued to rebound, with increases relative to Day 7 PI observed for all animals. Clinical pathology endpoints were compatible with DIC, renal dysfunction, dehydration, and liver damage.

In the surviving EBOV-exposed animal, plasma viral RNA, while detectable on Days 9 and 11 PI, had resolved with no detectable virus in samples obtained on Day 14 PI and later. Additionally, rash in this animal resolved by Day 11 PI, and decreases in behavioral activity (as assessed by responsiveness score) resolved by Day 11 PI. The surviving EBOV-exposed animal had a pattern of clinical pathology findings consistent with a lower grade of consumptive coagulopathy, reduced fluid loss, and reduced metabolic and tissue dysfunction compared to the other EBOV-exposed animals; coagulopathy and serum chemistry alterations resolved in this animal by Day 28 PI. Rebounds of lymphocyte, monocyte, platelets, and reticulocyte counts to levels exceeding baseline values occurred on Day 11 PI or later, and some of these parameters remained elevated through the end of the in-life phase.

### 3.10. Potential Triggers for Treatment

One of the primary goals of this study was to identify individual or combined manifestations of EVD that would be of potential use as triggers for initiation of treatment in evaluations of investigational therapeutics using this disease model.

To guide efforts to identify study endpoints that could potentially serve as a trigger, several factors were considered. A primary consideration is that the potential trigger must be specific to EBOV exposure (i.e., not observed in mock animals), such that it is distinguishable from any effects that could be attributed to experimental conditions. Second, any potential trigger would ideally be uniform, occurring in 100% of EBOV-exposed animals. Third, an ideal trigger should be measurable in an objective manner to eliminate subjective bias of the observer.

As previously noted, disease progression in this model occurs rapidly. The window for therapeutic intervention is narrow as animals succumb to the disease 7–10 days after virus exposure. Time-dependent changes in all collected parameters, from infected animals and mock-infected control animals, have been analyzed. Among those, body temperature, systemic viremia, physical activity, and clinical pathology endpoints are discussed in more detail as potential triggers.

#### 3.10.1. Body Temperature

In this study, body temperature monitoring occurred in real time via telemetry. Fever, defined as body temperature > 1.5 °C above baseline for longer than 2 h, occurred in 12 of 12 EBOV-exposed animals (100%) and in 0 of 6 of the Group 1 mock-exposed animals (0%; see Section 3.5). The onset of fever occurred by Day 3 PI (63.0–89.0 h after virus exposure) in 11 of 12 EBOV-exposed animals, with one remaining animal developing a fever on Day 4 PI (103.5 h; 4.3 days after virus exposure). Sustained elevations in body temperature were noted in all EBOV-exposed animals on Days 4–7 PI. Body temperature monitoring by telemetry provides an endpoint that is objective, specific, and uniform.

#### 3.10.2. Systemic Viremia

Plasma viral RNA was first detected in EBOV-exposed animals on Day 3 PI (*n* = 7); by Day 4 PI, all EBOV-exposed animals had detectable plasma viral RNA (see Section 3.3.1). While detection of viral RNA by PCR is a desirable trigger based on the objective nature of the endpoint and its uniform occurrence, there are several challenges to its use as a real-time individual animal treatment trigger. Processing and analysis of samples consumes substantial time, and additional delays could occur if reanalyses are needed (e.g., if assay results do not meet acceptance criteria). Additionally, given the rapid escalation of viral RNA concentration (Figure 3), a frequent sampling schedule would be needed to identify animals meeting a predefined trigger threshold. Although plasma viral RNA may not be feasible as an individual animal treatment trigger in this model, viral RNA data obtained retrospectively could be used, not as a trigger, but to confirm the systemic viral load/infection status in animals at the time a treatment is initiated.

#### 3.10.3. Animal Activity and Clinical Responsiveness

The activity of individual animals was assessed using two different methods: telemetric monitoring and assignment of a responsiveness score via direct observation of unanesthetized animals.

*Activity characterization* via *telemetry.* Using telemetry, animal activity data is derived from a signal produced on an automated and continuous basis by a surgically implanted transmitter that records movements (accelerations) in three dimensions. To analyze the telemetry-derived activity response, data were averaged over a time interval. These intervals included 6- and 12-h summaries, which began daily at 06:00 and ended at 18:00, consistent with the initiation of the daily light period in the animal rooms. Uniform, specific, and significant reductions in change-from-baseline activity values in EBOV-exposed animals occurred beginning on Day 4 PI in the 12-h interval (occurring 06:00–18:00), and animal activity generally progressively decreased thereafter through Day 8 PI (Figure 5).

*Responsiveness score.* In EBOV-exposed animals (Group 2), onset of clinically observed reduced behavioral activity and change in posture (i.e., assignment of a responsiveness score ≥ 1) first occurred on Day 5 PI; on this day, all EBOV-exposed animals were assigned a responsiveness score = 1 during at least one observation event (Figure 2). Responsiveness score, or equivalent assessments of clinical disease severity, may not be an optimal endpoint for use as a trigger because it is a subjective measure.

#### 3.10.4. Clinical Pathology Endpoints

A key aspect of any treatment-trigger approach that relies on clinical pathology data is the challenge of defining either an absolute or change-from-baseline response that is unequivocally associated with EVD progression in an individual animal. While it may be possible to define trigger thresholds on a study-specific basis, according to an animal’s baseline values, there is no clear regulatory or scientific consensus regarding the specific magnitude of change for any of these parameters that would be unequivocally associated with a diseased state. Nevertheless, because clinical pathology assessments were conducted from baseline blood samples obtained from EBOV-exposed animals prior to and again after exposure and because the sampling schedule was consistent between EBOV- and mock-exposed animals, it is possible to use the data from this study to show the times at which uniform, statistically significant, and specific alterations due to EBOV infection first occur, based on a daily sampling schedule.

**Figure A1:**
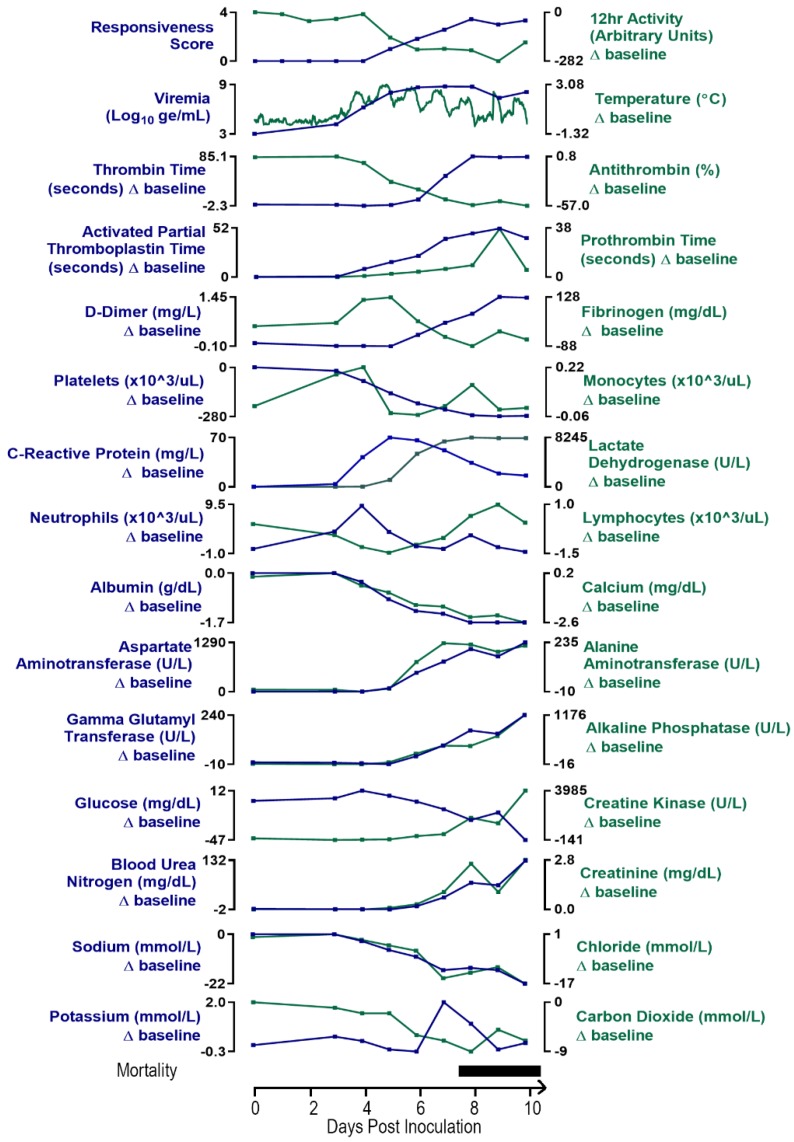
Clinical signs, viremia, and clinical pathology alterations in rhesus macaques exposed to IM EBOV. Data represent the mean for EBOV-exposed animals. Mean responsiveness score was derived from the individual animal maximum daily responsiveness score. The 12-h activity was derived from individual animal physical movements assessed via telemetry, averaged over the 12-h interval corresponding to the animal room light cycle (dark-cycle activity is not shown). For plasma viral RNA concentrations, EBOV RNA values below the LOD were imputed as 3.000 log_10_ ge/mL; values above the LOD but below the LLOQ were imputed as 4.903 log_10_ ge/mL. Temperature represents 30-min body temperature averages. X-axes are truncated to highlight responses occurring from the first sampling point through the acute disease phase. Error bars were omitted for clarity. The horizontal mortality bar indicates the range of timing in which animals succumbed.

Significant increases from baseline in CRP, fibrinogen, and neutrophil numbers were uniform in, and specific to EBOV-exposed animals beginning on Day 4 PI (Table 3 and Figure A1). Additional clinical pathology changes that were uniform and specific in EBOV-exposed animals include reduced lymphocyte counts and prolongation of the APTT. On Day 5 PI, significantly elevated levels of LDH and CRP and prolongations of APTT were specific to EBOV-exposed animals, as were decreased values of lymphocyte number and albumin, sodium, and antithrombin. Consistent with progressive worsening of disease signs, alterations in 12 clinical pathology parameters were uniformly observed in EBOV-exposed animals on Day 6, and 10 clinical pathology parameters were uniformly observed on Day 7 (however, instrument analysis issues precluded assessment of Na, K, and Cl on Day 7 PI).

## 4. Discussion

The primary objective of this study was to establish the disease characteristics, such as the following, in rhesus macaques exposed to EBOV via IM injection:

- Time from EBOV exposure to the onset of the manifestations of disease (e.g., signs, clinical and pathological features, laboratory parameters, extent of organ involvement, morbidity, and outcome)
- Time course and order of progression of the manifestations
- Frequency and severity of manifestations

After exposure to EBOV, 11 of 12 animals (91.7%) succumbed within 7–10 days (*n* = 10 were euthanized; *n* = 1 was discovered deceased). In animals that succumbed, the disease progressed rapidly, and key disease manifestations included viremia, fever, reduced physical activity, macular skin rash, systemic inflammation, coagulopathy, lymphocytolysis, renal tubular necrosis with mineralization, hepatocellular degeneration and necrosis, fluid loss, splenic fibrin deposition, and duodenal hemorrhage. The cumulative effect of multiple disease manifestations ultimately resulted in the rapid clinical decline of the nonsurviving EBOV-exposed animals. It is suspected that fluid loss, cytokine production, and clotting factor consumption contribute substantively to clinical decline of the NHP in addition to organ dysfunction. Fluid loss is suspected based on the observation of skin tenting and third space effusions in multiple animals; the microscopic hemorrhages consistently observed in nonsurvivors further demonstrate the propensity for vascular leakage during the course of disease. Although hepatic dysfunction contributes to the coagulopathy by reduced factor production, clotting factor consumption is likely significant given the widespread extent of fibrin deposition throughout multiple tissues, especially the splenic red pulp. The collected clinical pathology data evaluating clotting times throughout the disease course are consistent with this observation. In summary, it is the cumulative and aggregated effect of multiple disease manifestations that ultimately causes the rapid clinical decline of the infected NHP in this model.

### 4.1. Rhesus IM/EBOV Disease Compared to Human EVD

The results of this natural history study of the rhesus macaque IM/EBOV model confirm that this model reproduces the prominent features of fatal human EVD.

In comparing disease manifestations between humans and rhesus macaques, unavoidable objective limitations preclude direct comparison of certain disease signs/symptoms. First, clinical symptoms, such as headache and myalgia, cannot be assessed in animal studies; therefore, it is not possible to compare these symptoms between humans and rhesus macaques. Second, while detailed macroscopic and microscopic anatomic disease manifestations can be determined from post-mortem examinations and analyses in rhesus macaques, data from analogous post-mortem endpoints are rarely available from fatal human cases. The most direct comparisons can be made between objectively measurable clinical disease manifestations such as fever, changes in activity level, viral load, clinical signs, and clinical pathology laboratory values.

A comparison of key disease manifestations in the human EVD and rhesus IM/EBOV model is shown in Table 4.

Compared with that in humans, the progression of disease is more rapid in the IM/EBOV rhesus macaque model. The acute disease course in humans, in the absence of intervention, lasts approximately 1–3 weeks from EBOV exposure to death or recovery and typically results in <90% mortality. In rhesus macaques, the acute disease phase is compressed down to approximately one week from EBOV exposure and results in >90% mortality. Differences in the timing of disease progression and overall mortality rate may relate to the dose and route of EBOV exposure. Administration of a target dose of 1000 pfu by an IM route in rhesus macaques is intended to provide consistency in the experimental conditions among subjects within a particular trial and to produce disease conditions consistent with fatal human cases, to the extent possible. In contrast, EBOV exposure in humans is thought to occur typically by a mucosal route after contact with contaminated bodily fluids, and dose of EBOV is unknown.

In both humans and rhesus macaques, early signs of disease include fever, detectable plasma viral load, and clinical pathology findings indicative of systemic inflammation, lymphocytolysis, and coagulopathy. Gastrointestinal symptoms are more pronounced in humans. Rash and liver enzyme elevations occur in both humans and rhesus but appear earlier in the disease progression in humans. Late-stage disease in both humans and rhesus macaques is characterized by peak plasma viremia, liver and kidney dysfunction, and fluid loss.

### 4.2. Clinical vs. Nonclinical Efficacy of EVD Therapeutics

The PALM trial conducted in the DRC and the recent efficacy results obtained from human patients provide, for the first time, an opportunity to evaluate the extent to which efficacy results obtained through nonclinical assessments are predictive of clinical outcomes. Four investigational therapies—three monoclonal antibody-based products, mAb114, REGN-EB3, and ZMapp, and a small molecule nucleotide analog, remdesivir—were evaluated in the PALM trial, and for each of these, published reports describing the efficacy of regimens in the IM/EBOV rhesus macaque disease model are available [33,34,35,36]. The results achieved in the nonclinical model were of critical importance for identifying treatments with high therapeutic potential in EBOV-infected humans and were generally predictive of clinical efficacy. In humans, each of the investigational products improved the survival rates compared to untreated individuals, although the improvements varied between products.

One possible explanation for the differences in efficacy seen between the nonclinical model and in humans is that the severity of EBOV pathophysiology present in the rhesus macaques at Day 5 PI is not sufficiently representative of the spectrum with which humans presented during the outbreak. Further data are needed from nonclinical assessments of these investigational agents to determine whether efficacy results show greater consistency with reported human outcomes when treatments are initiated: (a) using a trigger associated with advanced disease; or (b) using a scheduled treatment initiation regimen beginning on Day 6 or 7 PI.

### 4.3. Regulatory Considerations and Future Role of the IM EBOV Rhesus Macaque Model of EVD

Conduct and completion of this natural history study was originally undertaken to characterize, in a manner compliant with regulatory expectations, the IM/EBOV rhesus macaque model to support EVD therapeutic product development under the Animal Rule. However, the demonstrated success of the PALM trial now shows that it was technically and logistically feasible to evaluate the efficacy of EVD investigational agents in humans during this outbreak. Nevertheless, the IM/EBOV rhesus model will likely continue to serve as a useful model by which to assess potential efficacy improvements associated with combinations or alternative formulations of mAb114, REGN-EB3, ZMapp, or remdesivir or to characterize efficacy of new products as part of the process by which candidates are selected for future clinical testing.

## Figures and Tables

**Table 1:** Serum infectious virus (log_10_ pfu/mL).

Day PI	Parameter	Mock	EBOV
−4	Mean (range)	<LOD (<LOD–<LOD)	<LOD (<LOD–<LOD)
	*n*	6	12
5	Mean (range)	<LOD (<LOD–<LOD)	3.45 (<LOD–5.61)
	*n*	6	12
7	Mean (range)	<LOD (<LOD–<LOD)	4.80 (DNQ–5.86)
	*n*	6	12
9	Mean (range)	<LOD (<LOD–<LOD)	4.12 (<LOD–5.57)
	*n*	6	4

DNQ, detectable not quantitative; LOD, limit of detection, 2.10 log_10_ pfu/mL; PI, post inoculation.

**Table 2:** Serum soluble glycoprotein concentration (ng/mL).

Day PI.	Mock-Exposed			EBOV-Exposed		
	Mean	Range	*n*	Mean	Range	*n*
4	<LLOQ	NA	6	636	52–2703	12
6	<LLOQ	NA	5	3148	1785–4211	12
7	NA	NA	NA	5461	3595–7326	2
8	NA	NA	NA	5647	2698–14096	5
9	NA	NA	NA	2400	NA	1
10	NA	NA	NA	2904	1883–3924	2
11	<LLOQ	NA	6	485	NA	1
21	<LLOQ	NA	6	<LLOQ	NA	1
28	<LLOQ	NA	6	<LLOQ	NA	1

LLOQ, lower limit of quantitation = 33 ng/mL; PI, post inoculation. Samples obtained for Days 7, 8, 9, and 10 PI were unscheduled events, occurring at the time of animal euthanasia.

**Table 3:** Timing of uniform, significant, and EBOV-specific clinical pathology changes.

		Days Post Infection			
		4	5	6	7
Hematology	Neutrophils (×10^3^/µL)	∧			
	Lymphocytes (×10^3^/µL)	∨	∨	∨	
	Platelets (×10^3^/µL)				∨
Coagulation	Antithrombin (%)		∨	∨	∨
	APTT (s)	∧	∧	∧	∧
	Fibrinogen (mg/dL)	∧			
	PT (s)			∧	∧
	Thrombin Time (s)			∧	∧
Serum Chemistry	AST (U/L)			∧	∧
	Albumin (g/dL)		∨	∨	∨
	CRP (mg/L)	∧	∧	∧	∧
	Calcium (mg/dL)			∨	∨
	Chloride (mmol/L)			∨	NA
	Lactate dehydrogenase (U/L)		∧	∧	∧
	Sodium (mmol/L)		∨	∨	NA

The listed parameters are those that, in EBOV-exposed animals, showed: (a) a statistically significant increase (∧) or decrease (∨) from baseline (*p* < 0.05); (b) uniform change from baseline in all EBOV-exposed animals (i.e., all increased or all decreased); and (c) no overlap with the range of change-from-baseline values of mock-exposed animals on the indicated day. Instrumentation errors precluded analysis of sodium and chloride on Day 7. APTT, activated partial thromboplastin time; AST, aspartate aminotransferase; CRP, C-reactive protein; NA, not available; PT, prothrombin time.

**Table 4:** Disease manifestations in humans and rhesus macaques.

Manifestations	Human	Rhesus (Based on Present Results)
**Incubation Period ^a^**	1–21 days ^b^ (more commonly 4–10 days ^c^)	2.5–4 days, mean 3 days
**Symptom Onset to Death**	6–16 days ^c^	4–7 days
**Mortality**	47–90% ^d^	91.7%
**Plasma Viremia**		
First Detectable	Approximately coincident with symptom onset ^e^	Coincident with symptom onset (3–4 days after infection)
Peak	5–7 days after symptom onset ^f^	2–4 days after symptom onset (5–7 days after infection)
**Clinical Disease Signs ^g^**		
Early	Fever Rash, diarrhea, vomiting	Fever Changes in activity
Late	Diffuse hemorrhage Shock Organ failure	Changes in responsiveness score Rash Abnormal motor function
**Clinical Pathology ^h^**	Systemic inflammation Coagulopathy Lymphocytolysis Liver dysfunction Renal dysfunction Fluid loss/dehydration	Systemic inflammation Coagulopathy Lymphocytolysis Liver dysfunction Renal dysfunction Fluid loss/dehydration
**Pathology ^i^**	Skin lesions (petechiae or ecchymoses) Lymphoid depletion of spleen and lymph nodes Necrosis in liver, kidney, spleen, gonads, gastrointestinal tract, and endocardium Diffuse hemorrhage	Skin lesions (macular skin rash) Lymphoid depletion of spleen and lymph nodes Hepatocellular and renal tubular necrosis Increased vascular permeability and leakage Micro- and macroscopic hemorrhage

^a^ In humans, the incubation period is defined as the time from presumed virus exposure to symptom onset. In the rhesus macaque natural history study, the incubation period is defined as the time from virus exposure to fever onset. ^b^ Source: [10]; ^c^ Source: [11]; ^d^ Source: [12]; ^e^ Sources: [13,14,15]; ^f^ Source: [16] ^g^ Data for humans are from the following sources: [11,17,18,19,20,21]. ^h^ Data for humans are from the following sources: [20,22,23,24,25,26,27,28,29,30,31]. ^i^ Data for humans are from the following sources: [12,32].

## Appendix A

**Table A1:** Schedule of study events.

Procedure	Day																							
	−7	−6	−5	−4	−3, −2, −1	0	1	2	3	4	5	6	7	8	9	10	11	12	13	14	15–20	21	22–28	Euthanasia Day, or Scheduled Termination for Survivors
Move to ABSL-4	X																							
Health Status Check				X																				
Continuous Telemetry Monitoring			X	X	X	X	X	X	X	X	X	X	X	X	X	X	X	X	X	X	X	X	X	X
Acclimation	X	X	X	X	X																			
Exposure						X																		
^1^ AM Observation (06:00–10:00)				X	X	X	X	X	X	X	X	X	X	X	X	X	X	X	X	X	X	X	X	X
AM Physical (anesthetized)				X		X														X		X		X
PCR				X		X			X	X	X	X	X		X		X			X		X		X
Hematology				X		X			X	X	X	X	X		X		X			X		X		X
Chemistry				X		X			X	X	X	X	X		X		X			X		X		X
Coagulation				X		X			X	X	X	X	X		X		X			X		X		X
Plaque Assay				X							X		X		X									X
Soluble GP ELISA						X				X		X					X					X		X
Anti-GP Antibody				X						X			X				X			X		X		X

^1^ Additional health status checks began when animals showed clinical signs (responsive score ≥ 1) and were discontinued when animals were recovering (responsiveness score = 0).
